# Effect of *Cichorium intybus* L. on the expression of hepatic NF-κB and IKKβ and serum TNF-α in STZ− and STZ+ niacinamide-induced diabetes in rats

**DOI:** 10.1186/s13098-016-0128-6

**Published:** 2016-02-13

**Authors:** Lotfollah Rezagholizadeh, Yasin Pourfarjam, Azin Nowrouzi, Manuchehr Nakhjavani, Alipasha Meysamie, Nasrin Ziamajidi, Peyman S. Nowrouzi

**Affiliations:** Department of Biochemistry, School of Medicine, Tehran University of Medical Sciences, Enghelab Avenue, Poursina Street, Tehran, Iran; Endocrinology and Metabolism Research Center, Vali-Asr Hospital, Tehran University of Medical Sciences, Tehran, Iran; Department of Community and Preventive Medicine, School of Medicine, Tehran University of Medical Sciences, Tehran, Iran; Department of Biochemistry, School of Medicine, Hamadan University of Medical Sciences, Hamadan, Iran

**Keywords:** Chicory, Diabetes type 2, Inflammatory cytokines, TNF-α, IKKβ, NF-κB

## Abstract

**Background:**

Inflammation is an early event in the development of diabetes type 2 (T2D). *Cichorium intybus* L. (chicory) possesses anti-inflammatory action. We compared the anti-inflammatory aspect of aqueous chicory seed extract (CSE) in early and late stage T2D in rats.

**Methods:**

Wistar albino rats were divided into nine final groups (*n* = *6*). Three main groups consisted of non-diabetic (Control), early stage diabetes (ET2D; niacinamide/streptozotocin, i.e., NIA/STZ), and late stage diabetes (LT2D; STZ). Within each main group, a subgroup was treated with CSE (125 mg/kg; i.p.); within each diabetic group (STZ and NIA/STZ) a subgroup received metformin (100 mg/kg; i.p.); another subgroup in STZ group received aspirin (120 mg/kg; oral). After 21 days, fasting blood glucose (FBS), insulin, and TNF-α level were measured in serum; IKKβ and NF-κB (p65) mRNA and protein expression were evaluated by real time PCR and Western blotting; p65 DNA binding activity was determined by ELISA, in liver tissue.

**Results:**

The mRNA and protein expression levels of IKKβ, and P65 genes increased in both stages of T2D (p < 0.01); CSE decreased their expression (p < 0.001, mRNAs; p < 0.05, proteins). The increased DNA-binding capacity of NF-κB (p < 0.0001) in diabetes was lowered by CSE (p < 0.001). The effect of CSE was limited to ET2D requiring insulin.

**Conclusions:**

The anti-inflammatory action of CSE is due to a direct modulation of cytokine expression. The dependency of chicory action on the presence of insulin indicates its usefulness in the early stages of diabetes and for the purpose of preventing and delaying diabetes onset.

**Electronic supplementary material:**

The online version of this article (doi:10.1186/s13098-016-0128-6) contains supplementary material, which is available to authorized users.

## Background

As a progressive disease, type 2 diabetes (T2D) may be divided into several stages based on the level of insulin in the serum. The “very early stage” of the disease, designated by us as VET2D, occurs when the beta cells show increased activity in an effort to compensate for the insulin resistance within the peripheral, such as muscle and adipose, tissues, and so there is hyperinsulinemia. This may be considered as the silent stage of T2D because patients are asymptomatic and the fasting blood sugar (FBS) stays within normal range although bouts of hyperglycemia may occur during postprandial states.

Sessions of high blood sugar can exert toxic effects, glucotoxicity, on the beta cells of the pancreas and lead to the “early stage” diabetes, ET2D, when there is insufficient production of insulin due to partial beta cell destruction, and FBS test may show slightly above normal. Healthy diet and shunning overnutrition, by remodeling the body, and regular physical activity, by sensitizing peripheral tissues toward insulin, can correct blood sugar levels at the onset of the disease. Drugs such as metformin and glibenclamide are used if diet changes and physical activity fail to lower blood sugar concentrations to normal range.

“Late stage” diabetes, LT2D, will finally occur when a total destruction of the pancreatic beta cells leads to a complete cessation of insulin secretion and the patients become dependent on regular insulin injections in addition to metformin. Constant unchecked high blood sugar will lead to “very late stage” diabetes, VLT2D, when patients suffer from severe complications, such as blindness, amputations, cardiovascular disease, renal complications, etc.

Major causes of insulin resistance are obesity and non-resolving low grade chronic inflammation and infection [1, 2]. During inflammation, small proteins, known as inflammatory cytokines, are produced by different cell types and secreted into the circulation [3]. Elevated levels of circulating inflammatory cytokines, including nuclear factor kappa-light-chain-enhancer of activated B cells (NF-κB) and tumor necrosis factor-alpha (TNF-α), in patients with postprandial hyperglycemia, may serve as predictors of the risk of type 2 diabetes [4].

Both TNF-α and NF-κB play important roles in insulin resistance and β-cell dysfunction and contribute to the pathogenesis of T2D, and lipid disorders [5–7]. NF-κB has modulating effect on the synthesis of many cytokines, including TNF-α, IK-1β, Il-6 and Il-8, due to having NF-κB binding sites in the enhancer region of the corresponding genes. In turn, TNF-α and IL-1 are potent activators of NF-κB. Binding of NF-κB to the DNA at the genes of TNF-α and other cytokines establishes a positive auto-regulatory loop and leads to amplification of inflammatory response and longer duration of chronic inflammation [8]. The ability of NF-κB to control multiple genes introduces the NF-κB signaling pathway as a potential drug target against inflammatory diseases [9].

*Cichorium intybus* L. (Chicory or Kasni) has been used in traditional medicine since the 17th century to treat digestive and hepatic disorders, as well as diabetes and inflammation. Modern research has been able to confirm the hypolipidemic, anti-oxidant, anti-inflammatory, atheroprotective, anti-hepatotoxic and antihyperglycemic roles of chicory and to some degree elucidate the underlying mechanisms of action [10–16]. These diverse biological effects result from an array of medicinally important compounds, such as alkaloids, inulin, sesquiterpene lactones, coumarins, vitamins, chlorophyll pigments, unsaturated sterols, flavonoids, saponins, tannins and polyphenols that are spread in all parts of the plant in different proportions [17]. Chicory, generally regarded as safe, has been implemented in several clinical trials with potential for delaying the onset of diabetes and management of osteoartheritis and cardiovascular disease [18–20].

Our studies with chicory have indicated its priority of use in early stages of diabetes when low amounts of insulin are present [21]. As chicory has anti-inflammatory property, we aimed to investigate this aspect of CSE in ET2D and LT2D in rats. We used metformin and aspirin as control drugs and for purpose of comparison.

Aspirin and metformin are examples of plant substances that have been commercialized and available for many decades [22, 23]. Aspirin, well-known for its anti-inflammatory effects, is used by over 60 % of diabetic patients for primary or secondary prevention of cardiovascular events [24, 25]. Metformin is the first-line pharmacological therapy for T2D, which is prescribed for over 120 million people worldwide [26].

## Methods

### Reagents

Streptozotocin (STZ) and niacinamide also called nicotinamide (NIA) were purchased from Sigma (USA). Citrate buffer (20 mM, pH 4.5) was prepared manually and autoclaved for sterilization. Bradford solution was prepared manually and filtered through Watman No 1 filter paper [27]. Metformin and aspirin pills (Chemidaru industrial company, Tehran, Iran) were purchased from a drug store. All other chemicals for Western blotting were analytical grade and from Sigma or Merck.

### Animals

Healthy adult male 8-week old Wistar albino rats weighing 190–260 g, were obtained from University of Tehran, Institute of Biochemistry and Biophysics and housed in standard and clean cages (2 per cage) under controlled environmental conditions at room temperature 22 ± 2 °C and 12-hour light–dark cycle with free access to a standard rat chow and water. Animal handling and treatment were performed in the Biochemistry Department of the School of Medicine, of Tehran University of Medical Sciences (TUMS). The study was ethically approved by the review board of TUMS.

### Plant extract, metformin and aspirin

The lyophilized CSE powder belonged to a previous study [21]. Metformin and aspirin pills were crushed manually. Certain amounts (mg) of the lyophilized CSE (125 mg/kg b.w., according to previous studies [21, 28]), metformin and aspirin (100 and 120 mg/kg, respectively, according to Sun et al. [29]) were weighed daily, in newly labeled Eppendorf vials, separately for each rat and according to weekly body weights measured by a digital balance (Sartorius, Germany). Citrate buffer (0.3 ml) was added, as vehicle, to each vial and vortex mixed immediately before administration.

### Diabetes induction

Early and late stage type 2 diabetes (ET2D and LT2D) were induced as previously explained [21]. Briefly, ET2D and LT2D were induced in overnight fasted rats by single intraperitoneal injections of streptozotocin (STZ, 55 mg/kg) or combination of STZ (55 mg/kg) and niacinamide (NIA, 200 mg/kg, 15 min later), dissolved in citrate buffer (0.3 ml). In creating ET2D, it is possible to inject NIA 15 min before or after STZ administration; in both occasions, NIA exerts partial protection against β-cytotoxic effect of STZ and leads to creation of milder form of diabetes [30–32]. Elevated FBS in blood from the tail vein (GlucoSure STAR, Taiwan), on days 4 and 10, following injection of STZ or STZ + NIA, was a confirmation of diabetes. FBS ranged between 140–220 mg/dl for the majority of rats on day 4 after injection of NIA+ STZ; therefore, rats were selected from among NIA + STZ-injected rats for ET2D groups when FBS ranged between 140–220 mg/dl on day 10 as well. FBS levels fell above 300 mg/dl in most of the STZ-injected rats on day 4, and therefore, rats were selected from STZ-injected rats, for LT2D groups when FBS was greater than 300 mg/dl on both days 4 and 10. Only rats with stable diabetes were included in the study; a few rats from STZ/NIA group with very high FBS and rats from both STZ and NIA/STZ groups, with normal FBS in the second occasion, day 10, were not included in the study. Normal rats, for the group Control, were injected with an equal volume of citrate buffer.

### Experimental groups and treatment

After confirmation of diabetes on day 10, control and diabetic animals were divided into nine groups of at least six rats, and treated by an appropriate regimen for 21 days, as follows; the total study time being 31 days. CSE and metformin were administered via i.p. at a dosage of 125 and 100 mg per kg body weight, respectively, suspended in 0.3 ml of citrate buffer. Aspirin, 120 mg per kg body weight, was given by gavage.

- *Control group* Non-diabetic controls treated with citrate buffer (0.3 ml).
- *CSE*-*control group* Non-diabetic controls treated with chicory seed extract.
- *NIA/STZ group* E-T2D rats treated with citrate buffer.
- *CSE*-*NIA/STZ group* E-T2D rats treated with chicory seed extract.
- *Met*-*NIA/STZ group* E-T2D groups treated with metformin.
- *STZ group* L-T2D rats treated with citrate buffer.
- *CSE*-*STZ group* L-T2D rats treated with chicory seed extract.
- *Met*-*STZ group* L-T2D groups treated with metformin.
- *Asp*-*STZ group* L-T2D groups that received aspirin.

### Weekly measurements and sample collection

During 21 days of daily treatment, FBS was measured in a drop of blood from the tail for three successive weeks. Likewise, body weights were recorded weekly.

### Sample collection

At the end of 21 day treatment time, the rats were transferred to metabolic cages at about 9 a.m. for 24 h urine collection with free access to water during this time; rat chow was removed at around 9 or 10 p.m. Without access to food until morning, the rats were in a state of fasting before sacrifice. The next day, FBS was determined, in a drop of blood from the tail and rats were anaesthetized with diethyl ether and blood samples collected by direct cardiac puncture; sera were separated and stored at −80 °C. The liver tissues were dissected, washed with cold phosphate buffer saline (PBS), submerged in liquid nitrogen, and stored in −80 °C. As we had only 8 metabolic cages in our animal room, we could handle only 8 animals per day, which we started with the animals with poorest health within the LT2D groups, continued with ET2D groups, and ended with controls.

### Insulin determination

Serum, collected as above at the end of the study, was used to measure insulin by Insulin (rat) ELISA kit (Abnova).

### Enzyme-linked immunosorbent assay of serum TNF-α

The measurement was performed by Tnf (rat) ELISA Kit according to the guidelines of the manufacturer (Abnova). Serum TNF-α was calculated according to a standard curve.

### Real-time PCR

Total RNA was extracted from liver tissue according to the manufacturer’s instructions (RNeasy Plus Mini Kit, Qiagen. Germany). Total RNA concentration was determined by spectrophotometric optical density measurement (NanoDrop 1000, Thermo Fisher Scientific; USA) at 260 and 280 nm. Reverse transcriptase reactions were then carried out with 1 µg of total RNA using the First Strand cDNA synthesis Kit (Thermoscientific, USA). Real-time PCR was performed on cDNA samples using SYBR Premix PCR kit (Takara, Japan). Primers for P65 (Cat. No QT00381227) IKKβ (Cat. No QT00188622), and β-actin (Cat. No QT00193473) as a reference gene were predesigned by QuantiTect Primer Assay (Qiagen, Germany). Samples were incubated at 95 °C for 5 min for an initial denaturation, followed by 40 cycles of denaturation at 95 °C for 5 s, and combined annealing and extension at 60 °C for 30 s. To confirm the amplification of specific transcript, melting curves were obtained at the end of each PCR run. The relative changes in gene expression were determined using CT and amplification of each sample. Real-time PCR was performed using Rotor-Gene Q (Qiagen, Germany).

### Western blotting

Whole tissue extracts were obtained by gently crushing 25 mg rat liver, for 1 min in a Potter–Elvehjem homogenizer (Thomas Scientific, Phila, USA), in 1 ml ice-cold RIPA buffer (50 mM Tris, pH 7.4, 150 mM NaCl, 5 mM EDTA, 1 % Triton X-100, 0.5 % sodium deoxycholate, 0.1 % SDS, 1 mM PMSF and 1 mM sodium orthovanadate) containing protease and phosphatase inhibitor cocktail. Each homogenized sample was triturated through a clean sterile insulin syringe once and left on ice for 30 min before centrifugation at 12,000×*g* at 4 °C for 20 min (Sigma 3–30 K). Some samples needed to be centrifuged a second time after careful collection and disposal of a fat layer on top. Proteins (60 µg, measured by Bradford method using BSA as standard) in 5× sample buffer were boiled for 5 min and resolved in 8 % SDS-PAGE, and immunoblotted (Mini Trans-Blot, Biorad, USA) [27]. The membranes were blocked in Phosphate Buffered Saline with 0.05 % Tween-20 (PBST) containing 5 % nonfat dry milk (Merck) at 4 °C overnight. After washing with PBST, membranes were incubated for 2 h at 37 °C with polyclonal rabbit anti-p65 antibody (1:1000) and polyclonal rabbit anti-IKKβ antibody (1:500) (Abcam, USA). After washing with PBST, membranes were incubated for 1 h at 37 °C with a horseradish peroxidase (HRP)-conjugated anti-rabbit IgG secondary antibody (1:20,000) (Abnova). The membranes were developed with the enhanced chemiluminescence (ECL) kit (Amersham Pharmacia Biotechnology). Protein levels were quantified by scanning densitometry using Image-J software.

### NF-κB (p65) DNA binding assay

DNA binding capacity of the NF-κB (p65) subunit in 20 µg of cell lysates was determined using an NF-κB (p65) enzyme-linked immunosorbent assay (ELISA)-based transcription factor assay kit (Abnova, Taiwan), where a specific double stranded DNA (dsDNA) sequence containing the NF-kB response element is immobilized onto the wells of a 96-well plate, according to the manufacturer’s protocol.

### Statistical analysis

Statistical analysis was performed by SPSS software (version 20). Normal distribution of continuous variables was checked by Kolmogrov-Smirnov test in each group. All variables in each group had normal distribution, so the comparison of continuous variables between different groups were carried out by one-way analysis of variance (ANOVA) followed by Scheffe and Dunnett T3 post hoc tests. For the changes of body weight and FBS values over time, within each rat group, comparison between time zero, day 10 and day 31, were performed by paired samples *t* test. Data were presented as the mean ± standard deviation (SD). The level of significance was considered 0.05.

## Results

### Body weights and blood sugar after induction of diabetes

T2D was characterized by constant hyperglycemia and weight loss. Without intervention, blood sugar levels continued to rise and body weights continued to fall, as expected (Table 1). The extent of weight loss was not significant in NIA/STZ-induced diabetic groups. Fasting blood glucose concentrations increased significantly to >300 mg/dl in STZ groups (LT2D), and ranged between 140–220 mg/dl in NIA/STZ groups (ET2D). The first part of the present study appears similar to a previous study from our laboratory [21]. In both studies, rats were distributed in a wide range of body weights (190–260 g). In the 2012 study, we distributed the rats in different groups so to have similar average body weights in all groups which led to a wide standard deviation from the start. In the present study, however, we placed the rats with closest body weights in each group to obtain small standard deviations for the average weights. The rats with lowest body weight were assigned for Control. CSE was able to significantly lower FBS (p < 0.001) and cause an increase in body weight (p = 0.032) in ET2D rats in confirmation of our previous results (Table 1; Additional file 1) [21].

**Table 1:** Body weight and fasting blood sugar levels upon L-T2D and E-T2D induction and after a 21-day treatment with chicory, metformin and aspirin

Parameter	Non-diabetic (control)		ET2D			LT2D			
	Control	CSE-control	NIA/STZ	CSE- NIA/STZ	Met-NIA/STZ	STZ	CSE-STZ	Met-STZ	Asp-STZ
Weight (g ± SD)									
Time zero	185.1 ± 4.3	192.8 ± 2.5	241.2 ± 22.1	236.8 ± 30.0	264.3 ± 23.5	248.1 ± 16.7^aa^	240.4 ± 35.1	260.7 ± 10.4	256.8 ± 17.2
Diabetes induction, start of treatment (day 10)	192.1 ± 9.2	194.6 ± 6.6	226.8 ± 23.1	248.6 ± 21.1	252.5 ± 26.4	239.1 ± 8.4^aaa^	232.0 ± 32.5	226.3 ± 16.6^***^	238.6 ± 26.3
End of 21 day treatment time (day 31)	232.1 ± 20.8^††,§§^	217.8 ± 12.6^††,§^	229.4 ± 15.8	258.3 ± 14.3^§^	258.5 ± 19.1	222.8 ± 16.3^§^	215.4 ± 32.6^§^	231.3 ± 13.3^†††^	234.8 ± 18.8^††^
FBS (mg/dl ± SD)									
Time zero	119.5 ± 5.7	111.8 ± 7.5	95.2 ± 6.3^aa^	99.0 ± 5.8^aa^	98.8 ± 9.2^a^	99.3 ± 12.3	108.2 ± 8.4	97.4 ± 5.1^aa^	98.5 ± 3.9^aa^
Diabetes induction, start of treatment (day 10)	111.3 ± 7.3^**^	107.0 ± 11.6	202.2 ± 8.3^aaa,***^	153.0 ± 12.61^bb,***^	222.4 ± 9.7^aaa,***^	401.5 ± 32.6^aaa,***^	346.8 ± 19.5^aaa***^	341.0 ± 16.5^aaa***^	307.8 ± 7.4^aaa,c,***^
End of 21 day treatment time (day 31)	107.8 ± 11.1^†^	103.5 ± 6.1	305.4 ± 88.4^††^	82.3 ± 10.2^b,††,§§§^	307.4 ± 156.7^†^	398.6 ± 25.5^bbb,†††^	365.4 ± 44.5^†††^	371.0 ± 67.9^†††^	454.8 ± 72.1^†††,§§^

*, †, and § indicate, respectively, statistically significant differences between time zero vs. day 10; time zero vs. day 31; and day 10 vs. day 31 (single, p < 0.05; double, p < 0.01; triple, p < 0.001). Weights on day 0 for Met-NIA/STZ, STZ, CSE-STZ, and Asp-STZ were significantly higher than control and CSE-control (p < 0.01); for Met-STZ vs. Control and CSE-control, p < 0.001. FBS levels on day 10 in all diabetic groups were significantly higher than Control and CSE-control (p < 0.001)

^a^Significant differences between any column vs. Control. ^b^Significant difference between NIA/STZ vs. CSE-NIA/STZ. ^c^Significant difference between Asp-STZ vs. STZ (single letters indicate p < 0.05; double letters p < 0.01; and triple letter p < 0.001)

### Insulin levels in serum

**Fig. 1:**
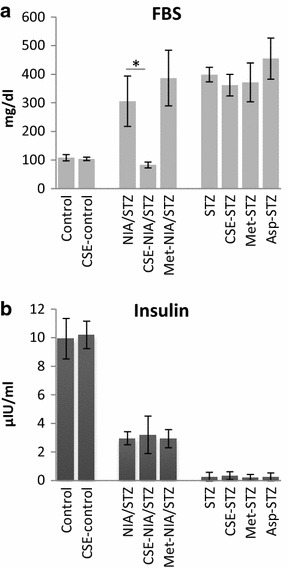
Serum FBS (**a**) and insulin levels (**b**) at the end of study time (day 31). Serum insulin levels decreased significantly in NIA/STZ (p = 0.001) and STZ (*p* < 0.0001) diabetic groups relative to Control and CSE-control. Neither CSE nor metformin or aspirin treatment significantly improved serum levels of insulin in treated groups. The decrease by CSE of glucose levels in CSE-NIA/STZ group relative to NIA/STZ group, however, was marginally significant (*p* = 0.057). Data are presented as mean ± SD. It may be worth to mention that FBS levels in CSE-NIA/STZ group on day 31 was significantly lower compared to day 10 (Table 1, *p* < 0.001)

Serum insulin levels decreased about 70 % in NIA/STZ-induced diabetes (p = 0.001) and about 97 % in STZ-induced diabetes (*p* < 0.001), compared with non-diabetic control rats. Although insulin levels were measured at the end of study time (day 31) we can still reasonably conclude that there was a significantly measurable amount of insulin in the NIA/STZ groups all through the study time and no insulin was present in the STZ groups (NIA/STZ group vs. STZ group, p < 0.001). Therefore, we categorized the NIA/STZ-induced diabetes as ET2D, and the STZ-induced diabetes as LT2D. The status of fasting blood sugar and blood insulin are shown in Fig. 1a, b.

### TNF-α in serum

**Fig. 2:**
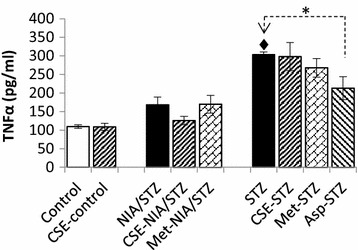
Effect of CSE on TNF-α. Serum TNF-α levels increased significantly in STZ group compared with Control and CSE-control. Aspirin decreased serum TNF-α in Asp-STZ group compared with STZ; *p < 0.001; ^♦^ *p* < 0.001 versus controls. Values are mean ± SD of all subjects in each group

TNF-α was elevated after diabetes induction (53 % in ET2D, p = 0.084; and 177 % in L-T2D, p < 0.001) as compared to controls. CSE and metformin did not significantly lower the level of TNF-α (Fig. 2). Aspirin led to about 30 % decrease in serum TNF-α concentration in Asp-STZ group compared with STZ group (*p* = 0.003). While aspirin might be expected to improve TNF-α level in ET2D, not tested in this study, CSE certainly was unable to lower TNF-α level in LT2D.

### Protein and mRNA expression in liver

Real-time PCR and Western-blot analysis revealed low expression of IKKβ and NF-κB (p65) in the control groups (Control and CSE-control). After diabetes induction, the elevation in mRNA levels of both IKKβ and NF-κB (p65) genes was greater for LT2D (STZ-group) than ET2D (NIA/STZ group).

**Fig. 3:**
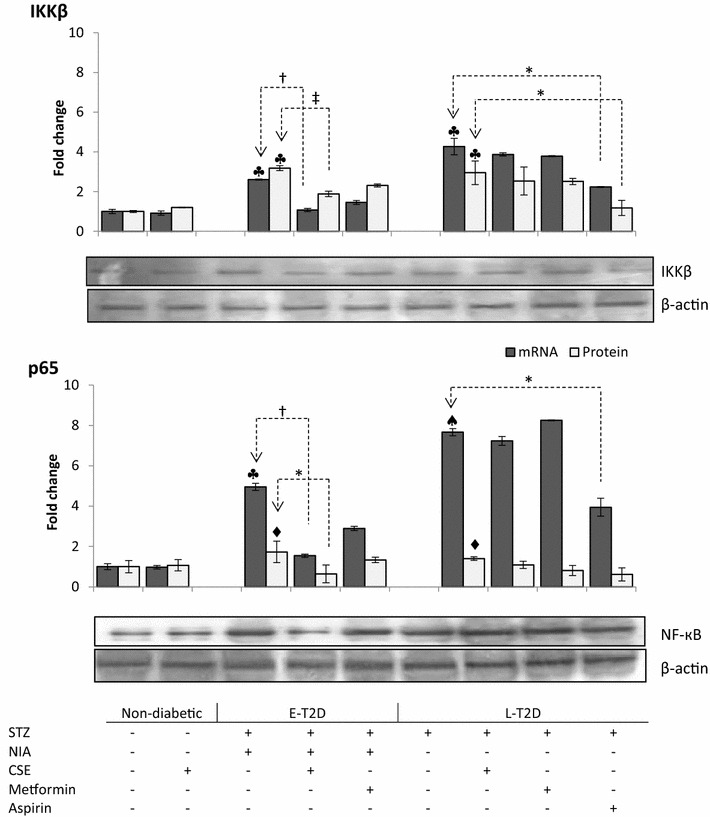
The expression levels of mRNA and protein for IKKβ, and NF-κB (p65) genes. The mRNA and protein expression levels for both genes were up-regulated in STZ and NIA/STZ groups relative to controls. CSE treatment led to down-regulation of the mRNA and protein levels of both genes in CSE-NIA/STZ group in comparison to NIA/STZ group. Metformin caused an insignificant decrease in Met-NIA/STZ (met-NIA/STZ vs. NIA/STZ: *p* = 0.22 and *p* = 0.06 for IKKβ mRNA and protein; *p* = 0.07 and *p* = 0.08 for p65 mRNA and protein, respectively). The mRNA levels of IKKβ, and p65 genes were effectively down-regulated in Asp-STZ compared to STZ; although aspirin significantly decreased the protein levels of IKKβ, it was not able to effectively lower the p65 protein level. *p < 0.01; ^‡^ *p* < 0.05; ^†^ *p* < 0.001. ♣, ♦ and ♠ represent *p* < 0.01, *p* < 0.05 and *p* < 0.001, respectively, versus non-diabetic controls. *Dotted lines* show comparisons between treated and their respective untreated groups. Data are expressed as mean ± SD of 3 duplicate experiments for real time. Four replicates were used for protein quantification

Treatment with CSE and metformin down-regulated the expression rates of mRNAs and proteins corresponding to IKKβ and NF-κB (p65) in ET2D rats which was significant in case of CSE (CSE-NIA/STZ and Met-NIA/STZ vs. NIA/STZ, *p* < 0.01 and *p* = 0.06, respectively). The expression rate of mRNA and protein levels for IKKβ was lowered significantly by aspirin (Asp-STZ vs. STZ, *p* < 0.01) whereas the p65 protein did not seem to be affected (Fig. 3). Western blot analysis on four members of each group is shown in Additional file  2.

### DNA-binding activity of P65 in liver

**Fig. 4:**
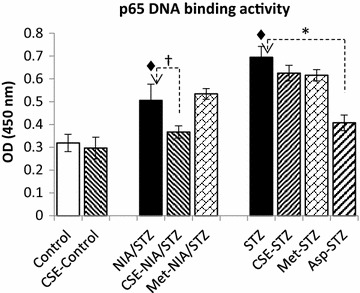
Effects of chicory extract on P65 DNA-binding activity. Liver tissue cell lysates with equal concentration of protein (20 µg; determined by Bradford) were used in this experiment. Optical density (OD) of samples was measured at 450 nm and OD changes was compared in all groups. P65 activity increased significantly in NIA/STZ and STZ groups compared with Control and CSE-control. Treatment with CSE significantly decreased P65 activity in CSE-NIA/STZ group compare with NIA/STZ group. Treatment with aspirin decreased it in Asp-STZ group when compared with STZ group. Metformin did not affect the P65 activity. Data are expressed as mean ± SD. *p < 0.0001; ^†^ *p* < 0.001; ^♦^ *p* < 0.0001 versus Control

The DNA-binding activity of the NF-kB p65 factor strongly increased in the liver tissues of diabetic rats (NIA/STZ and STZ groups vs. Control, *p* < 0.001). Treatment with CSE significantly decreased P65 DNA-binding activity (CSE-NIA/STZ vs. NIA/STZ, *p* < 0.001). In ET2D, metformin did not improve the P65 DNA-binding activity after 21 days, compared with NIA/STZ group. There were no significant changes in LT2D groups treated with CSE or metformin. Treatment with aspirin significantly decreased P65 DNA-binding activity (Asp-STZ vs. STZ, *p* < 0.001) (Fig. 4).

## Discussion

Despite a wide variety of oral hypoglycemic drugs, search for new herbs and nutraceuticals continues due to several limitations of existing drugs [33].

Plant extracts contain many small bioactive molecules with selective activities in natural proportions that can exhibit acute effects on many of the features of T2D, such as oxidative stress, inflammation, and hyperglycemia, by acting on multiple targets and via different mechanisms, simultaneously [34]. Some plant components have strong free radical scavenging capabilities that modulate oxidative stress, others induce detoxification and antioxidant enzymes, and yet others have revealed to act via inhibition of signal-induced cytokine expression [35, 36].

The results show that inflammatory cytokines are upregulated in diabetes in both early and late stages; and suggest that CSE (125 mg/kg) is efficient in lowering blood sugar compared to metformin (100 mg/kg dosage) and aspirin (120 mg/kg) and that it possesses anti-inflammatory effect. For the second time we show that, interestingly, CSE is effective only during ET2D and not LT2D stages and therefore, its effectiveness seems dependent on the presence of insulin.

Based on data in literature and our own experience, CSE may be acting through several mechanisms to lower inflammation in diabetic rats. First, as hyperglycemia can induce activation of NF-κB through advanced glycation end products [37], the anti-inflammatory effect of CSE may be secondary to glucose lowering effect, which might also explain to some degree the rationale behind the ineffectiveness of CSE in LT2D. We have previously determined that CSE is able to lower blood sugar in LT2D rats when insulin and CSE are given together; however, to help understand the insulin dependency of anti-inflammatory action of CSE, STZ and NIA/STZ rats treated with insulin and chicory should be provided in the future studies [38].

In line with previous reports, the findings suggest that the anti-inflammatory effect of CSE might be linked with reduced expression of proinflammatory cytokines, such as TNF-α and NF-κB, in ET2D rats [39]. TNF-α is produced in the early stages of inflammation and controls the production of other cytokines. It is expressed as a 26-kDa cell surface transmembrane protein that is cleaved to a 17-kDa soluble form when activated [40]. Both the membrane bound and the soluble forms of TNF-α can bind TNFR1 (TNF receptor type 1) and TNFR2 (TNF receptor type 2). After binding to its receptors, TNFα can lead to insulin resistance via inhibiting insulin receptor (IR) signaling [7]. Therefore, less TNF-α would mean less contact with its receptors and more insulin sensitization. CSE did not significantly decrease TNF-α in serum in ET2D. It is not clear if longer duration of treatment would be able to show more significant TNF-α lowering effect, which could then be considered as the second mechanism.

Binding of TNF-α to its cell surface receptors can also lead to activation of canonical NF-κB pathway. In nonstimulated cells, NF-κB dimers stay in the cytoplasm through interaction with inhibitory proteins, I kappa Bs (IκBs). After stimulation with proinflammatory cytokines such as TNF-α, the sequence of events in canonical NF-κB pathway involves rapid activation of multicomponent protein kinase, the I kappa B kinase (IKK), leading to phosphorylation of two critical serines in the N-terminal regulatory domain of IκBs, especially IκBα. Phosphorylated IκBα is recognized by a specific E3 ubiquitin ligase complex and undergoes polyubiquitination which targets it for rapid degradation by the 26S proteasome. NF-κB dimers, which are spared from degradation, translocate to the nucleus to bind DNA and activate inducible expression of several genes [41]. Regulation of NF-κB activities by a range of endogenous and exogenous regulators, including oxidants/antioxidants from plants, can occur via targeting any one of the above specific steps in the NF-κB pathway.

The results of the present study showed that the amount of IKKβ mRNA and protein increased in diabetes and CSE was able to decrease the mRNA and protein levels  of IKKβ. Likewise, the amount of P65 that represents NF-κB heterodimer increased in diabetic rats; CSE attenuated the expression of mRNA and protein of p65. Finally, the DNA-binding capacity of NF-κB which had increased in diabetes was also lowered by CSE.

Besides having direct effect on the expression of mRNA and protein of NF-κB (p65), CSE might have effects on each of the steps of the cascade, including phosphorylation, ubiquitination and degradation of IκBα, which we did not determine. However, by targeting the canonical NF-κB cascade at IKK at the upstream, CSE would be expected to bring about a partial reduction in all the steps of the cascade via reducing the amount of initial substrate for each step.

In addition to indirectly controlling the amount of NF-κB, and the cytoplasmic signaling events, phenolic antioxidant components of CSE may be capable of affecting the functions of NF-κB in the nucleus. Some CSE components, such as caffeic acid, may directly inhibit binding of NF-κB with its DNA binding sequence [35] and other components capable of undergoing reduction–oxidation cycling may interfere by DNA binding of NF-κB indirectly; for example, via interacting with IRS-3 or targeting a redox sensitive protein factor in the nucleus [36, 42]. Inhibition of IκBα degradation, or an inhibition of DNA-binding via alkylation of p65 subunit of IκBα, have also been suggested for CSE’s ability to inhibit NF-κB activation [43]. Targeting different steps of canonical NF-κB cascade can be categorized as the third mechanism of action.

We have previously determined that CSE acts as a PPARα activator [44]. Therefore, in addition to acting via its free radical scavenging elements, CSE may be able to inhibit the high glucose-induced production of cellular ROS by inhibiting NADPH oxidase activity [45]. Both aspirin and chicory are inhibitors of cyclooxygenase 1 (COX-1) and COX-2 [23]. However, the inability of CSE to influence inflammatory markers in LT2D suggested a dependence of CSE’s anti-inflammatory activity on insulin, or different mechanisms of action for aspirin and chicory.

Regarding the effects of metformin, it is well established that metformin has evident anti-diabetic effects in T2D, both clinically and experimentally. In addition to anti-diabetic action, metformin seems to have other useful properties as extending life span and acting against cancer. Metformin’s inability to reduce TNF-α in serum was in contrast with recent reports that measured TNF-α in isolated human monocytes and mice liver. However, higher dose of metformin (250 mg/kg) was used to reduce TNF-α levels in blood and liver of the mice [46], and the suppression of TNF-α production in human monocytes, depended on metformin concentration [47]. The quality and dose of metformin pill from Chemidaru Industrial Company was checked against metformin pill from Merck using HPLC, and was estimated to contain an average of 19.4 % ± 5.2 less metformin per pill (Additional file 3) [48]. As stated earlier, we chose the metformin dosage according to Sun et al. who found 100 mg/kg metformin to be effective in lowering blood sugar in STZ-induced diabetic rats with hyperinsulinemia, labeled as VET2D by us [46]; but clearly the effect of metformin as an insulin sensitizer should depend on the stage of diabetes in terms of insulin levels in the body. In the present study, the rats had ET2D, which is a more advanced stage of the disease with lower insulin levels and it may not be surprising that higher doses of metformin would probably be more effective. Therefore, the inability to see much effect for metformin, in this study, may be due to low dosage.

The mode of drug administration may play a role as well. In the present study, metformin and chicory extract were administered via intraperitoneal injection. As metformin exists in highest concentration in the portal vein, intestinal absorption may be the most convenient and proper way for metformin to access the liver and the gut, the main sites of action for metformin [26, 49].

Diet, weight control, physical activity, patient education and pharmacological approaches, are ways to manage T2D. Our results show that in early stage diabetes (ET2D), where insulin secretion from pancreas is significantly low, CSE performs better than metformin or aspirin in normalizing blood sugar; and better than metformin and as good as aspirin in reducing inflammation. Aspirin can possibly target the markers of inflammation in all stages of diabetes and probably in other inflammatory conditions, without major effect on blood sugar. CSE could not significantly improve any of the late stage diabetes conditions; however, if CSE’s mechanism of anti-inflammatory and anti-hyperglycemic activities is mediated by insulin, it may benefit patients with LT2D who inject insulin.

The role for insulin in enabling CSE to lower the markers of inflammation deserves more investigation. Failure of CSE, in the present study, to improve insulin levels in NIA/STZ animals was in agreement with the results of some studies [18, 28] and in contrast to others [21, 50]. Given that insulin levels fluctuate till stable manifestation of diabetes, an interim analysis of insulin in ET2D animals would have been informative to understanding the temporal effects of CSE, and a more definitive conclusion about whether or not CSE can stimulate more insulin secretion from the remaining beta cell of the pancreas.

The results stress on the importance of medical nutrition therapy (MNT) in prevention of inflammation-based diseases [51] and highlight the view that other than the dosage of medicines, that should be tailor-made for every patient, different medications may be required at different stages of the disease.

## Additional files

10.1186/s13098-016-0128-6 Body weights and fasting blood sugar (FBS) levels upon ET2D and LT2D induction and during 21-day treatment with CSE, metformin and aspirin; same data as Table 1.
10.1186/s13098-016-0128-6 Western blot analysis on 4 members of each group.
10.1186/s13098-016-0128-6 Evaluation of metformin. The HPLC system consisted of PLATIN Blue (KNAUER, Germany) with a PDA detector. The wavelength was set at 233 nm. The column was Nucleosil-100, C-18 (250 4.6 mm, 5 nm). The software was EZChrom Elite. The mobile phase comprised 0.01 M potassium dihydrogen orthophosphate (adjusted to pH 4.5 with glacial acetic acid) and acetonitrile (60:40, v/v). Analyses were run at a flow-rate of 1.0 ml/min and the samples were quantified using peak area. Initial weight of each 500 mg pill: Merck, 516.63 mg; and Chemidaru, 582.50 mg. 2.5, 5, 10, 50, and 100 mg of the powdered pills were dissolved in 1 ml of deionized H_2_O. Merck metformin dissolved completely in water, whereas metformin from Chemidaru consisted of insoluble part. Injection volumes were 5 µl; each sample was injected only once. The corresponding peaks for 10 mg/ml sample and the resulting standard curves for two metformin brands are compared in graphs.

## Abbreviations

CSE: *Cichorium intybus* seed extract
ECL: enhanced chemiluminescence detection
ET2D: early stage diabetes type 2
FBS: fasting blood sugar
IκB: I kappa B
IKK: I kappa B kinase
Il-6: interleukin 6
LT2D: late stage diabetes type 2
NF-κB: nuclear factor kappa-light-chain-enhancer of activated B cells
NIA/STZ: niacinamide/streptozotocin
NIA: niacinamide (nicotinamide)
PBST: phosphate buffered saline (PBS) solution with the detergent tween 20
PMSF: phenylmethylsulfonyl fluoride
PPARα: peroxisome proliferator-activated receptor alpha
PVDF: polyvinylidene fluoride
RIPA buffer: radioImmunoprecipitation Assay buffer
STZ: streptozotocin
T2D: diabetes type 2
TNF-α: tumor necrosis factor-alpha
Transcription factor p65: nuclear factor NF-kappa-B p65 subunit
VET2D: very early stage diabetes type 2
VLT2D: very late diabetes type 2
