# Supplementary material for: Effect of Cichorium intybus L. on the expression of hepatic NF-κB and IKKβ and serum TNF-α in STZ− and STZ+ niacinamide-induced diabetes in rats
Source: Diabetol Metab Syndr. 2016 Feb 13;8:11. doi: 10.1186/s13098-016-0128-6 (PMC4752748; doi:10.1186/s13098-016-0128-6)
Supplement: Supplementary file 1 — 10.1186/s13098-016-0128-6 Body weights and fasting blood sugar (FBS) levels upon ET2D and LT2D induction and during 21-day treatment with CSE, metformin and aspirin; same data as Table 1. [file 13098_2016_128_MOESM1_ESM.docx]

**Supplement 1**
