# Supplementary figures and images for: Effect of Cichorium intybus L. on the expression of hepatic NF-κB and IKKβ and serum TNF-α in STZ− and STZ+ niacinamide-induced diabetes in rats
Source: Diabetol Metab Syndr. 2016 Feb 13;8:11. doi: 10.1186/s13098-016-0128-6 (PMC4752748; doi:10.1186/s13098-016-0128-6)

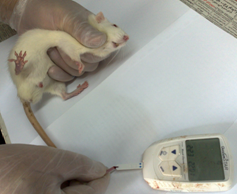

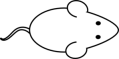

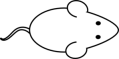

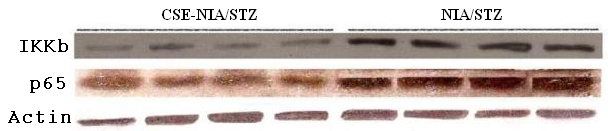

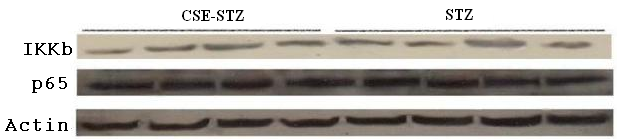
**Supplement 2**

NIA + STZ

STZ

FBS > 300 mg/dl

FBS ≈ 140-220 mg/dl


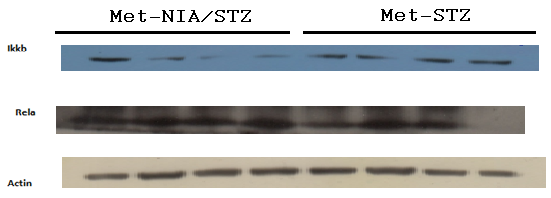


*

*


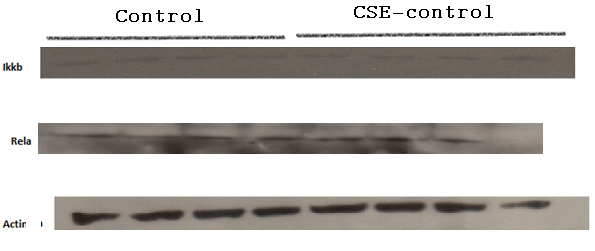


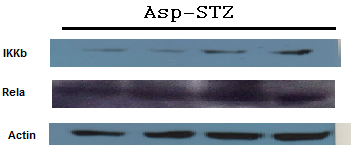

Supplement: Supplementary file 2 — 10.1186/s13098-016-0128-6 Western blot analysis on 4 members of each group. [file 13098_2016_128_MOESM2_ESM.docx]

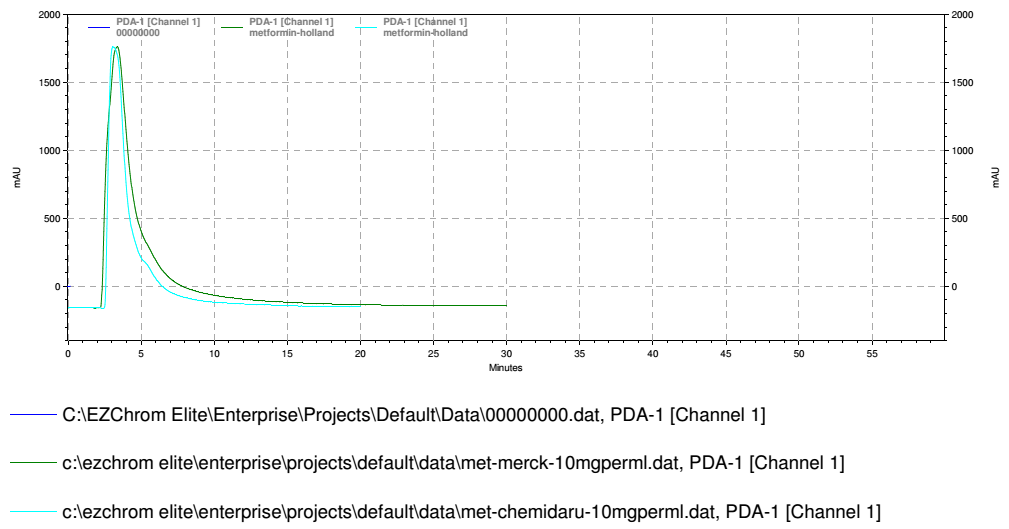


**Supplement 3**

Supplement: Supplementary file 3 — 10.1186/s13098-016-0128-6 Evaluation of metformin. The HPLC system consisted of PLATIN Blue (KNAUER, Germany) with a PDA detector. The wavelength was set at 233 nm. The column was Nucleosil-100, C-18 (250 4.6 mm, 5 nm). The software was EZChrom Elite. The mobile phase comprised 0.01 M potassium dihydrogen orthophosphate (adjusted to pH 4.5 with glacial acetic acid) and acetonitrile (60:40, v/v). Analyses were run at a flow-rate of 1.0 ml/min and the samples were quantified using peak area. Initial weight of each 500 mg pill: Merck, 516.63 mg; and Chemidaru, 582.50 mg. 2.5, 5, 10, 50, and 100 mg of the powdered pills were dissolved in 1 ml of deionized H2O. Merck metformin dissolved completely in water, whereas metformin from Chemidaru consisted of insoluble part. Injection volumes were 5 µl; each sample was injected only once. The corresponding peaks for 10 mg/ml sample and the resulting standard curves for two metformin brands are compared in graphs. [file 13098_2016_128_MOESM3_ESM.docx]
